# Development and application of a next-generation-sequencing (NGS) approach to detect known and novel gene defects underlying retinal diseases

**DOI:** 10.1186/1750-1172-7-8

**Published:** 2012-01-25

**Authors:** Isabelle Audo, Kinga M Bujakowska, Thierry Léveillard, Saddek Mohand-Saïd, Marie-Elise Lancelot, Aurore Germain, Aline Antonio, Christelle Michiels, Jean-Paul Saraiva, Mélanie Letexier, José-Alain Sahel, Shomi S Bhattacharya, Christina Zeitz

**Affiliations:** 1 INSERM, U968, Paris, F-75012, France; 2 CNRS, UMR_7210, Paris, F-75012, France; 3 UPMC Univ Paris 06, UMR_S 968, Department of Genetics, Institut de la Vision, Paris, F-75012, France; 4 Centre Hospitalier National d'Ophtalmologie des Quinze-Vingts, INSERM-DHOS CIC 503, Paris, F-75012, France; 5 UCL-Institute of Ophthalmology, London, UK; 6 IntegraGen SA, Genopole CAMPUS 1 bat G8 FR-91030 EVRY France; 7 Fondation Ophtalmologique Adolphe de Rothschild, Paris, France; 8 Académie des Sciences-Institut de France, 75006 Paris, France; 9 Department of Celular Therapy and Regenerative Medicine, Andalusian Molecular Biology and Regenerative Medicine Centre (CABIMER), Isla de Cartuja, Seville, Spain

**Keywords:** NGS, retinal disorders, diagnostic tool.

## Abstract

**Background:**

Inherited retinal disorders are clinically and genetically heterogeneous with more than 150 gene defects accounting for the diversity of disease phenotypes. So far, mutation detection was mainly performed by APEX technology and direct Sanger sequencing of known genes. However, these methods are time consuming, expensive and unable to provide a result if the patient carries a new gene mutation. In addition, multiplicity of phenotypes associated with the same gene defect may be overlooked.

**Methods:**

To overcome these challenges, we designed an exon sequencing array to target 254 known and candidate genes using Agilent capture. Subsequently, 20 DNA samples from 17 different families, including four patients with known mutations were sequenced using Illumina Genome Analyzer IIx next-generation-sequencing (NGS) platform. Different filtering approaches were applied to identify the genetic defect. The most likely disease causing variants were analyzed by Sanger sequencing. Co-segregation and sequencing analysis of control samples validated the pathogenicity of the observed variants.

**Results:**

The phenotype of the patients included retinitis pigmentosa, congenital stationary night blindness, Best disease, early-onset cone dystrophy and Stargardt disease. In three of four control samples with known genotypes NGS detected the expected mutations. Three known and five novel mutations were identified in *NR2E3, PRPF3, EYS, PRPF8, CRB1, TRPM1 *and *CACNA1F*. One of the control samples with a known genotype belongs to a family with two clinical phenotypes (Best and CSNB), where a novel mutation was identified for CSNB. In six families the disease associated mutations were not found, indicating that novel gene defects remain to be identified.

**Conclusions:**

In summary, this unbiased and time-efficient NGS approach allowed mutation detection in 75% of control cases and in 57% of test cases. Furthermore, it has the possibility of associating known gene defects with novel phenotypes and mode of inheritance.

## Background

Inherited retinal disorders affect approximately 1 in 2000 individuals worldwide [1]. Symptoms and associated phenotypes are variable. In some groups the disease can be mild and stationary such as in congenital stationary night blindness (CSNB) or achromatopsia (ACHM), whereas other disorders are progressive leading to severe visual impairment such as in rod-cone dystrophies, also known as retinitis pigmentosa (RP) or cone and cone-rod dystrophies. The heterogeneity of these diseases is reflected in the number of underlying gene defects. To date more than 150 genes have been implicated in different forms of retinal disorders http://www.sph.uth.tmc.edu/Retnet/home.htm and yet in a significant proportion of patients the disease causing mutation could not be identified, suggesting additional novel genes that remain to be discovered. Furthermore, recent studies have outlined that distinct phenotypes can be related to the dysfunction of the same gene [2-4]. Furthermore, there may be additional phenotype-genotype associations that are still not recognized. The state-of-the-art phenotypic characterization including precise family history and functional as well as structural assessment (i.e. routine ophthalmic examination, perimetry, color vision, full field and multifocal electroretinography (ERG), fundus autofluorescence (FAF) imaging and optical coherence tomography (OCT)) allows targeted mutation analysis for some disorders. However, in most cases of inherited retinal diseases, similar phenotypic features can be due to a large number of different gene defects.

Various methods can be used for the identification of the corresponding genetic defect. All these methods have advantages and disadvantages. Sanger sequencing is still the gold-standard in determining the gene defect, but due to the heterogeneity of the disorders it is time consuming and expensive to screen all known genes. Mutation detection by commercially available APEX genotyping microarrays (ASPER Ophthalmics, Estonia) [5,6] allows the detection of only known mutations. In addition, a separate microarray has been designed for each inheritance pattern, which tends to escalate the costs especially in simplex cases, for which inheritance pattern cannot be predetermined. Indirect methods with single nucleotide polymorphism (SNP) microarrays for linkage and homozygosity mapping are also powerful tools, which has proven its reliability in identifying novel and known gene defects [7-12]. However, in case of homozygosity mapping the method can only be applied to consanguineous families or inbred populations. To overcome these challenges, we designed a custom sequencing array in collaboration with a company (IntegraGen, Evry, France) to target all exons and part of flanking sequences for 254 known and candidate retinal genes. This array was subsequently applied through NGS to a cohort of 20 patients from 17 families with different inheritance pattern and clinical diagnosis including RP, CSNB, Best disease, early-onset cone dystrophy and Stargardt disease.

## Methods

### Clinical investigation

The study protocol adhered to the tenets of the Declaration of Helsinki and was approved by the local Ethics Committee (CPP, Ile de France V). Informed written consent was obtained from each study participant. Index patients underwent full ophthalmic examination as described before [13]. Whenever available, blood samples from affected and unaffected family members were collected for co-segregation analysis.

### Previous molecular genetic analysis

Total genomic DNA was extracted from peripheral blood leucocytes according to manufacturer's recommendations (Qiagen, Courtaboeuf, France). DNA samples from some patients with a diagnosis of RP were first analyzed and excluded for known mutations by applying commercially available microarray analysis (arRP and adRP ASPER Ophthalmics, Tartu, Estonia). In some cases, pathogenic variants in *EYS, C2orf71, RHO, PRPF31, PRPH2 *and *RP1 *were excluded by direct Sanger sequencing of the coding exonic and flanking intronic regions of the respective genes [13-17]. Conditions used to amplify *PRPH2 *can be provided on request.

### Molecular genetic analysis using NGS

A custom-made SureSelect oligonucleotide probe library was designed to capture the exons of 254 genes for different retinal disorders and candidate genes according to Agilent's recommendations (Table 1). These genes include 177 known genes underlying retinal dysfunction (http://www.sph.uth.tmc.edu/retnet/sum-dis.htm, October 2010, Table 1) and 77 candidate genes associated with existing animal models and expression data (Table 2). The eArray web-based probe design tool was used for this purpose https://earray.chem.agilent.com/earray. The following parameters were chosen for probe design: 120 bp length, 3× probe-tiling frequency, 20 bp overlap in restricted regions, which were identified by the implementation of eArray's RepeatMasker program. A total of 27,430 probes, covering 1177 Mb, were designed and synthesized by Agilent Technologies (Santa Clara, CA, USA). Sequence capture, enrichment, and elution were performed according to the manufacturer's instructions (SureSelect, Agilent). Briefly, three μg of each genomic DNA were fragmented by sonication and purified to yield fragments of 150-200 bps. Paired-end adaptor oligonucleotides from Illumina were ligated on repaired DNA fragments, which were then purified and enriched by six PCR cycles. 500 ng of the purified libraries were hybridized to the SureSelect oligo probe capture library for 24 h. After hybridization, washing, and elution, the eluted fraction underwent 14 cycles of PCR-amplification. This was followed by purification and quantification by qPCR to obtain sufficient DNA template for downstream applications. Each eluted-enriched DNA sample was then sequenced on an Illumina GAIIx as paired-end 75 bp reads. Image analysis and base calling was performed using Illumina Real Time Analysis (RTA) Pipeline version 1.10 with default parameters. Sequence reads were aligned to the reference human genome (UCSC hg19) using commercially available software (CASAVA1.7, Illumina) and the ELANDv2 alignment algorithm. Sequence variation annotation was performed using the IntegraGen in-house pipeline, which consisted of gene annotation (RefSeq), detection of known polymorphisms (dbSNP 131, 1000 Genome) followed by mutation characterization (exonic, intronic, silent, nonsense etc.). For each position, the exomic frequencies (homozygous and heterozygous) were determined from all the exomes already sequenced by IntegraGen and the exome results provided by HapMap project.

**Table 1:** Known retinal disease genes

*Number*	*Gene name*
1	***ABCA4***

2	***ABCC6***

3	***ADAM9***

4	***AHI1***

5	***AIPL1***

6	***ALMS1***

7	***ARL6***

8	***ARMS2***

9	***ATXN7***

10	***BBS10***

11	***BBS12***

12	***BBS2***

13	***BBS4***

14	***BBS5***

15	***BBS7***

16	***BBS9***

17	***BEST1***

18	***C1QTNF5***

19	***C2***

20	***C2orf71***

21	***C3***

22	***CA4***

23	***CABP4***

24	***CACNA1F***

25	***CACNA2D4***

26	***CC2D2A***

27	***CDH23***

28	***CDH3***

29	***CEP290***

30	***CERKL***

31	***CFB***

32	***CFH***

33	***CHM***

34	***CLN3***

35	***CLRN1***

36	***CNGA1***

37	***CNGA3***

38	***CNGB1***

39	***CNGB3***

40	***CNNM4***

41	***COL11A1***

42	***COL2A1***

43	***COL9A1***

44	***CRB1***

45	***CRX***

46	***CYP4V2***

47	***DFNB31***

48	***DMD***

49	***DPP3***

50	***EFEMP1***

51	***ELOVL4***

52	***ERCC6***

53	***EYS***

54	***FAM161A***

55	***FBLN5***

56	***FSCN2***

57	***FZD4***

58	***GNAT1***

59	***GNAT2***

60	***GPR98***

61	***GRK1***

62	***GRM6***

63	***GUCA1A***

64	***GUCA1B***

65	***GUCY2D***

66	***HMCN1***

67	***HTRA1***

68	***IDH3B***

69	***IMPDH1***

70	***IMPG2***

71	***INPP5E***

72	***INVS***

73	***IQCB1***

74	***JAG1***

75	***KCNJ13***

76	***KCNV2***

77	***KLHL7***

78	***LCA5***

79	***LRAT***

80	***LRP5***

81	***MERTK***

82	***MFRP***

83	***MKKS***

84	***MKS1***

85	***MTND1***

86	***MTND6***

87	***MT-AP6***

88	***MTND2***

89	***MTND5***

90	***MTND4***

91	***MYO7A***

92	***NDP***

93	***NPHP1***

94	***NPHP3***

95	***NPHP4***

96	***NR2E3***

97	***NRL***

98	***NYX***

99	***OAT***

100	***OFD1***

101	***OPA1***

102	***OPA3***

103	***OPN1LW***

104	***OPN1MW***

105	***OPN1Sw***

106	***OTX2***

107	***PANK2***

108	***PAX2***

109	***PCDH15***

110	***PCDH21***

111	***PDE6A***

112	***PDE6B***

113	***PDE6C***

114	***PDE6G***

115	***PDZD7***

116	***PEX1***

117	***PEX2***

118	***PEX7***

119	***PGK1***

120	***PHYH***

121	***PITPNM3***

122	***PRCD***

123	***PROM1***

124	***PRPF3***

125	***PRPF31***

126	***PRPF8***

127	***PRPH2***

128	***RAX2***

129	***RB1***

130	***RBP3***

131	***RBP4***

132	***RD3***

133	***RDH12***

134	***RDH5***

135	***RGR***

136	***RGS9***

137	***RGS9BP***

138	***RHO***

139	***RIMS1***

140	***RLBP1***

141	***ROM1***

142	***RP1***

143	***RP1L1***

144	***RP2***

145	***RP9***

146	***RPE65***

147	***RPGR***

148	***RPGRIP1***

149	***RPGRIP1L***

150	***RS1***

151	***SAG***

152	***SDCCAG8***

153	***SEMA4A***

154	***SLC24A1***

155	***SNRNP200***

156	***SPATA7***

157	***TEAD1***

158	***TIMM8A***

159	***TIMP3***

160	***TLR3***

161	***TLR4***

162	***TMEM126A***

163	***TOPORS***

164	***TREX1***

165	***TRIM32***

166	***TRPM1***

167	***TSPAN12***

168	***TTC8***

169	***TTPA***

170	***TULP1***

171	***UNC119***

172	***USH1C***

173	***USH1G***

174	***USH2A***

175	***VCAN***

176	***WFS1***

177	***ZNF513***

**Table 2:** Candidate genes for retinal disorders

*Number*	*Gene name*	*Reason*	*References*
1	***ADCY1***	diff. Expression rd1 mouse	Chalmel et al., manuscript in preparatiom

2	***ANKRD33***	diff. Expression rd1 mouse	Chalmel et al., manuscript in preparatiom

3	***ANXA2***	Promotion of choroidal neovascularization	[36]

4	***ARL13B***	Cilia protein, mutations lead to Joubert Syndrome	[37]

5	***BMP7***	Regulation of Pax 2 in mouse retina	[38]

6	***BSG***	-	Thierry Leveillard personal commmunication

7	***CAMK2D***	diff. Expression rd1 mouse	Chalmel et al., manuscript in preparatiom

8	***CCDC28B***	Modifier for BBS	[39,40]

9	***CLCN7***	Cln7-/- mice severe osteopetrosis and retinal degeneration	[41]

10	***COL4A3***	Alport syndrome, with eye abnormalities	[42,43]

11	***COL4A4***	Alport syndrome, with eye abnormalities	[42,44]

12	***COL4A5***	Alport syndrome, with eye abnormalities	[42,45]

13	***CUBN***	-	Personal communication Renata Kozyraki

14	***CYP1B1***	glaucoma	[46]

15	***DOHH***	diff. Expression rd1 mouse	Chalmel et al., manuscript in preparatiom

16	***DSCAML1***	diff. Expression rd1 mouse	Chalmel et al., manuscript in preparatiom

17	***ESRRB***	diff. Expression rd1 mouse	Chalmel et al., manuscript in preparatiom

18	***FIZ1***	Interactor of *NRL*	[47]

19	***GJA9***	diff. Expression rd1 mouse	Chalmel et al., manuscript in preparatiom

20	***GNAZ***	diff. Expression rd1 mouse	Chalmel et al., manuscript in preparatiom

21	***GNGT1***	diff. Expression rd1 mouse	Chalmel et al., manuscript in preparatiom

22	***GPR152***	diff. Expression rd1 mouse	Chalmel et al., manuscript in preparatiom

23	***HCN1***	diff. Expression rd1 mouse	Chalmel et al., manuscript in preparatiom

24	***HEATR5A***	diff. Expression rd1 mouse	Chalmel et al., manuscript in preparatiom

25	***HIST1H1C***	Expressed in retina	Expression databases

26	***IMPG1***	diff. Expression rd1 mouse	Chalmel et al., manuscript in preparatiom

27	***INSL5***	diff. Expression rd1 mouse	Chalmel et al., manuscript in preparatiom

28	***KCNB1***	diff. expression rd1 mouse	Chalmel et al., manuscript in preparatiom

29	***KCTD7***	Expressed in retina	Expression databases

30	***LASS4***	diff. expression rd1 mouse	Chalmel et al., manuscript in preparatiom

31	***LRIT2***	diff. expression rd1 mouse	Chalmel et al., manuscript in preparatiom Rd1 mouse

32	***LRP2***	-	Personal communication Renata Kozyraki

33	***MAB21L1***	diff. expression Rd1 mouse	Chalmel et al., manuscript in preparatiom

34	***MAP2***	diff. expression rd1 mouse	Chalmel et al., manuscript in preparatiom

35	***MAS1***	Degeneration of cones due to expression of *Mas1*	[48]

36	***MAST2***	diff. expression rd1 mouse	Chalmel et al., manuscript in preparatiom

37	***MPP4***	diff. expression rd1 mouse	Chalmel et al., manuscript in preparatiom

38	***MYOC***	glaucoma	[49]

39	***NDUFA12***	diff. expression rd1 mouse	Chalmel et al., manuscript in preparatiom

40	***NEUROD1***	BETA2/NeuroD1 -/- mouse: photoreceptor degeneration	[50]

41	***NOS2***	glaucoma	[51]

42	***NXNL1***	Rod-derived cone viability factor	[52]

43	***NXNL2***	Rod-derived cone viability factor 2	[53]

44	***OPN1MW2***	Cone opsin, medium-wave-sensitive2	[54]

45	***OPTN***	glaucoma	[55]

46	***PFKFB2***	diff. expression rd1 mouse	Chalmel et al., manuscript in preparatiom

47	***PIAS3***	Rod photoreceptor development	[56]

48	***PKD2L1***	Diff. expression in human retinal detachment	Delyfer et al. 2011 submitted

49	***PLEKHA1***	Age-related macular degeneratiom	[57]

50	***PPEF2***	diff. expression rd1 mouse	Chalmel et al., manuscript in preparatiom

51	***RAB8A***	Interacts with RPGR, role in cilia biogenesis and maintenance	[58]

52	***RABGEF1***	diff. expression rd1 mouse	Chalmel et al., manuscript in preparatiom

53	***RCVRN***	diff. expression rd1 mouse	Chalmel et al., manuscript in preparatiom

54	***RGS20***	diff. expression rd1 mouse	Chalmel et al., manuscript in preparatiom

55	***RNF144B***	diff. expression rd1 mouse	Chalmel et al., manuscript in preparatiom

56	***RORB***	Rod photoreceptor development in mice	[59]

57	***RXRG***	Retinoic acid receptor, highly expressed in the eye	Expression databases

58	***SGIP1***	diff. expression rd1 mouse	Chalmel et al., manuscript in preparatiom

59	***SLC16A8***	Altered visual function in ko-mice	[60]

60	***SLC17A7***	diff. expression rd1 mouse	Chalmel et al., manuscript in preparatiom

61	***STAM2***	diff. expression rd1 mouse	Chalmel et al., manuscript in preparatiom

62	***STK35***	diff. expression rd1 mouse	Chalmel et al., manuscript in preparatiom

63	***STX3***	diff. expression rd1 mouse	Chalmel et al., manuscript in preparatiom

64	***SV2B***	diff. expression rd1 mouse	Chalmel et al., manuscript in preparatiom

65	***TBC1D24***	diff. expression rd1 mouse	Chalmel et al., manuscript in preparatiom

66	***THRB***	Essential for M-cone development in rodents	[61]

67	***TMEM216***	Cilia protein, mutations lead to Joubert and Meckel syndrome	[62]

68	***TMEM67***	Cilia protein, mutations lead to Joubert	[63]

69	***TRPC1***	diff. expression rd1 mouse	diff. expression Rd1 mouse

70	***UHMK1***	diff. expression rd1 mouse	diff. expression Rd1 mouse

71	***VSX1***	Stimulator for promoter NXNL1	[64]

72	***VSX2***	Stimulator for promoter NXNL1	[64]

73	***WDR17***	diff. expression rd1 mouse	diff. expression Rd1 mouse

74	***WDR31***	diff. expression Nxnl1-/- mouse	[65]

75	***WISP1***	diff. expression rd1 mouse	Chalmel et al., manuscript in preparatiom

76	***XIAP***	Protects photoreceptors in animal models of RP	[66]

77	***ZDHHC2***	diff. expression Rd1 mouse	Chalmel et al., manuscript in preparatiom

### Investigation of annotated sequencing data

**Figure 1:**
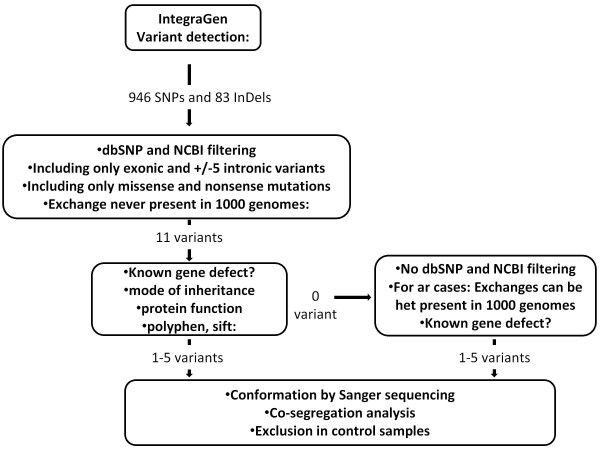
**Flow chart of variant analysis**. IntegraGen provided the results in form of excel tables. For each sample on average 946 SNPs and 83 inDels were detected, of which 11 represent missense, nonsense or putative splice site mutations, which were absent in dbSNB, NCBI and 1000 genome databases. Of those 1-5 variants were predicted to be pathogenic. In case where none of the variants were predicted to be pathogenic, dbSNB, NCBI and 1000 genome databases were included to detect mutations referenced with an rs-number. Co-segregation analysis was performed in families with putative pathogenic variants.

We received the annotated sequencing data in the form of excel tables. On average 946 SNPs and 83 insertions and deletions were identified for each sample (Figure 1). By using the filtering system, we first investigated variants (nonsense and missense mutations, intronic variants located +/- 5 apart from exon), which were absent in dbSNP and NCBI databases http://ncbi.nlm.nih.gov/. In the absence of known gene defects or putative pathogenic variants (see below) in the first step, we selected known genes, which were previously clinically associated including variants present in dbSNP and NCBI databases (Figure 1). Each predicted pathogenic variant was confirmed by Sanger sequencing.

### Assessment of the pathogenicity of variants

Following criteria were applied to evaluate the pathogenic nature of novel variations identified by NGS: 1) stop/frameshift variants were considered as most likely to be disease causing; 2) co-segregation in the family; 3) absence in control samples; 4) for missense mutations amino acid conservation was studied in the UCSC Genome Browser http://genome.ucsc.edu/ across species from all different evolutionary branches. If the amino acid residue did not change it was considered as "highly conserved". If a different change was seen in fewer than five species and not in the primates then it was considered as "moderately conserved" and if a change was present in 5-7, it was considered as "weakly conserved", otherwise the amino acid residue was considered as "not conserved", 5) pathogenicity predictions with bioinformatic tools (Polyphen: *Poly*morphism *Phen*otyping, http://genetics.bwh.harvard.edu/pph/ and SIFT: Sorting Intolerant From Tolerant, http://blocks.fhcrc.org/sift/SIFT.html) if at least one of the program predicted the variant to be possibly damaging, it was considered to be pathogenic; 6) presence of the second mutant allele in the case of autosomal recessive inheritance. Mutations were described according to the HGVS website http://www.hgvs.org/mutnomen. In accordance with this nomenclature, nucleotide numbering reflects cDNA numbering with +1 corresponding to the A of the ATG translation initiation codon in the reference sequence. The initiation codon is codon 1. The correct nomenclature for mutation was checked applying Mutalyzer http://www.lovd.nl/mutalyzer/.

## Results

The overall sequencing coverage of the captured regions was 98.4% and 90.4% for a 1× and a 10× coverage respectively. The overall sequencing depth was > 120×. The number of reference and variant sequences detected by NGS, reflected the correct zygosity state of the variant; on average if 50% of the sequences represented the variant, then a heterozygous state was called, while if 100% of the sequences represented the variant, then a homozygous or hemizygous state was annotated by IntegraGen.

### Validation of the novel genetic testing tool for retinal disorders

To validate the novel genetic testing tool for retinal disorders, we used four DNA samples from families, in which we had previously identified different types of mutations by Sanger sequencing: one 1 bp duplication and one 1 bp deletion in *PRPF31 *and missense mutations in *TRPM1 *and *BEST1 *(Table 3). Three of the four mutations were detectable by NGS, whereas the deletion in *PRPF31 *was not identified. To validate if this was due to a technical problem of deletion detection in general or low coverage at this position, the sequencing depth was investigated in detail. Indeed the coverage at this position reflected by the mean depth was only ~1-6 for all samples. This indicates that although the coverage in general was very good, specific probes used here need to be redesigned to improve the capture for specific exons.

**Table 3:** Patients with known mutations used to validate the novel genetic approach for retinal disorders

Index	Phenotype	Gene	Mutation	Allele State	Read reference NGS	Read variant NGS	Mutation detected by NGS	Mean depth
CIC00034, F28	adRP	*PRPF31*	c.666dup p.I223YfsX56	het	11	13	yes	21.3-22.5

CIC00140, F108	adRP	*PRPF31*	c.997delG p.E333SfsX5	het	-	-	no	5.0-5.2

CIC00238, F165	arCSNB	*TRPM1*	c.1418G > C p.R473P	homo	0	38	yes	36.7

CIC00707, F470	Best and adCSNB see Table 5	*BEST1*	c.73C > T p.R25W	het	40	38	yes	99.4

### Detection of known and novel mutations

**Figure 2:**
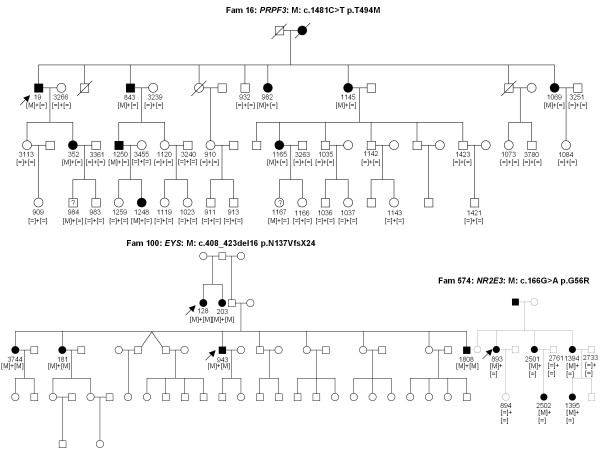
**Detection of known mutations by NGS in 254 retinal genes**. The index patient 19 of family 16 with adRP revealed the p.T494M mutations in *PRPF3*, which co-segregates with the phenotype. Two family members never clinically investigated from the last generation (984 and 1167 carrying a question mark) were reported to be not affected but carried the mutation. They may develop the phenotype at a later stage. In addition variability of the phenotype of this mutation was documented [35]. Two patients, 128 and 943 of family 100 with arRP from Jewish origin revealed the known *EYS *mutation p.N137VfsX24, which was found in all screened affected family members. The index patient 893 of family 574 showed the previously described NR2E3 p.G56R mutation, which co-segregated with the phenotype.

**Figure 3:**
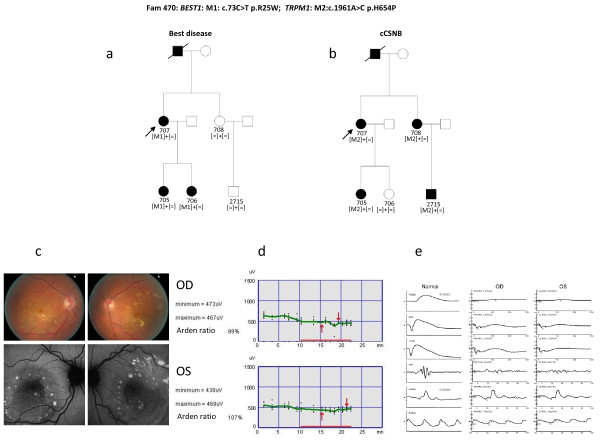
**Best disease and CSNB co-segregating in one family**. a) Sanger and NGS detected in all patients with Best disease a *BEST1 *mutation. b) NGS detected in all patients with a cCSNB phenotype a novel *TRPM1 *mutation. c) Fundus colour photographs (above) and fundus autofluorescence (below) of patient 707 showing multiple yellow deposits within the posterior pole which are hyper autofluorescent d) Electro-oculogram of patient 707 showing no slight rise after illumination in keeping with the diagnosis of Best disease e) Full Field Electroretinogram of patient 707 showing ON-bipolar cell pathway dysfunction in keeping with the diagnosis of cCSNB.

**Figure 4:**
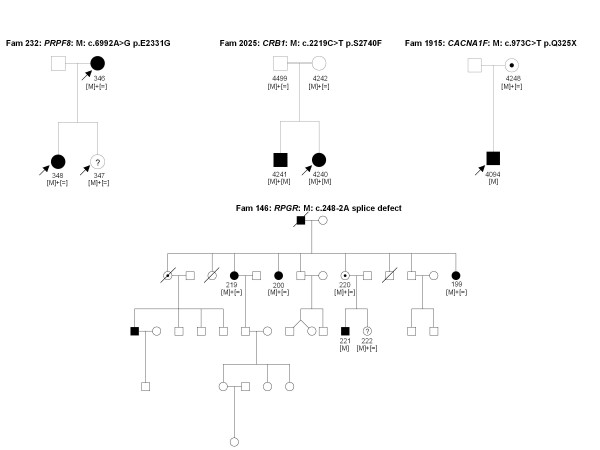
**Detection of novel mutations using NGS in 254 retinal genes**. Novel mutations in *PRPF8, CRB1, RPGR *and *CACNA1F *co-segregated in affected and asymptomatic carriers with the adRP, arRP, x-linked dominant and X-liked icCSNB phenotypes respectively. Asymptomatic individuals are marked with a question mark.

Some of the patients from the 14 families with no known gene defect were previously excluded for known mutations using microarray analysis and by Sanger sequencing in the known genes *EYS, C2orf71, RHO, PRPF31, PRPH2 *and *RP1*. Other samples were never genetically investigated. In four DNA samples known mutations were detected (Table 4) from three different families with autosomal dominant (ad) or recessive (ar) RP. All mutations co-segregated with the phenotype (Figure 2). In seven samples, novel mutations in known genes were identified. These mutations co-segregated with the phenotype from five different families with adCSNB, x-linked incomplete CSNB, adRP, arRP and x-linked RP (Table 5, Figures 3 and 4). One of the cases from these five families was also used as a control for Best disease carrying a known *BEST1 *mutation (Table 3). In addition to the Best phenotype, ERG-responses of this patient resembled those of complete CSNB, i.e. showing selective ON-bipolar pathway dysfunction. This phenotype was independent of the Best phenotype (Figure 3). The most likely disease causing mutation detected by NGS was a novel heterozygous *TRPM1 *mutation (Table 4, Figure 3).

**Table 4:** Detection of known mutations by using the novel genetic approach for retinal disorders

Index	Phenotype	Pre-screening	Gene	Mutation	Allele State	Read reference NGS	Read variant NGS	Reference	Mutation verified by Sanger and co-segregation
**CIC00019, F16**	adRP	Linkage, *RHO, PRPF31, PRPH2, RP1*	***PRPF3***	**c.1481C > T** **p.T494M**	het	25	22	[67]	yes

**CIC0000893, F574**	adRP	*RHO, PRPF31, PRPH2, RP1*	***NR2E3***	**c.166G > A** **p.G56R**	het	5	3	[68]	yes

**CIC000128, F100**	arRP, consang.	-	***EYS***	**c.408_423del p.N137VfsX24**	homo	-	179	[13,69]	yes

CIC0000943, F100	arRP, consang	-	*EYS*	c.408_423del p.N137VfsX24	homo	0	193	[13,69]	yes

**Table 5:** Detection of novel mutations by using the novel genetic approach for retinal disorders

Index	Phenotype	Pre-screening	Gene	Mutation	Allele State	Read reference NGS	Read variant NGS	Mutation verified by Sanger and co-segregation	Conservation	Polyphen	Sift
**CIC00707**, **F470**	adCSNB and Best see Table 3	*RHO, PDE6B, GNAT1*	***TRPM1***	**c.1961A > C** **p.H654P**	het	39	38	yes	moderately conserved	possibly damaging	tolerated

**CIC000348, F232**	adRP, mild	*RHO, PRPF31, PRPH2, RP1*, adRP chip	***PRPF8***	**c.6992A > G** **p.E2331G**	het	13	10	yes	moderately conserved	possibly damaging	affect protein function

CIC000346, F232	adRP	-	*PRPF8*	c.6992A > G p.E2331G	het	5	9	yes	moderately conserved	possibly damaging	affect protein function

CIC000347, F232	as adRP	-	*PRPF8*	c.6992A > G p.E2331G	het	15	17	yes	moderately conserved	possibly damaging	affect protein function

**CIC04240**, **F2025**	arRP, consang., detailed clinic in [70]	*RS1*	***CRB1***	**c.2219C > T** **p.S740F**	homo	2	194	yes	highly conserved	probably damaging	affect protein function

**CIC00199**, **F146**	adRP or x-linked RP with affected carrier	*RHO, PRPF31, PRPH2, RP1*, adRP chip	***RPGR***	**c.248-2A > G** **splice defect**	hetero	30	22	yes	conserved splice site	n.a.	n.a.

**CIC04094**, **F1915**	icCSNB	-	***CACNA1F***	**c.973C > T** **p.Q325X**	hemi	0	28	yes	n.a.	n.a.	n.a.

### Unsolved cases

**Figure 5:**
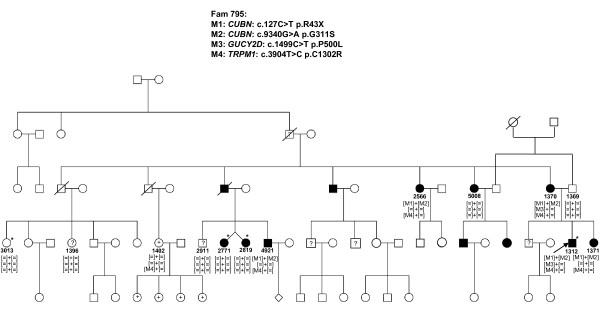
**Detection of novel mutation by using NGS in 254 retinal genes**. Family 795 reveals autosomal dominant cone dystrophy with post-receptoral defects. Four putative disease causing mutations were investigated on the basis of co-segregation. However, none of them co-segregated in all affected family members with the phenotype and thus are not considered to be disease causing. Individuals marked with a star were clinically investigated, patients with a question mark are asymptomatic and patients with a plus sign show high myopia.

In six of the 14 families with Stargardt disease, adRP, adCD with postreceptoral defects, arRP, early onset arCD with macrocephaly and mental retardation described in affected sister and x-linked cCSNB, the disease associated mutations remain to be elucidated or validated (Table 6, Figure 5).

**Table 6:** Patients with unsolved genotype and unlikely disease causing mutations

Index	Phenotype	Pre-screening	Gene	Mutation	Allele State	Read reference NGS	Read variant NGS	Mutation verified by Sanger and co-segregation	Comment
**CIC03282**, **F1388**	Stargardt	*ABCA4 *microarray	***ABCA4***	**c.1268A > G** **p.H423R**	het	77	61	yes	but reported as polymorphism [71]
				**c.6764G > T** **p.S2255I** **no additional variants in lower covered exons**	het	2	7	yes	but reported as polymorphism [72]

			***CFH***	**c.3482C > A** **p.P1161Q**	het	77	52	yes	conserved, probably damaging
				**c.1204C > T** **p.H402Y**	het	94	87	yes	AMD

**CIC01269, F761**	adRP	*-*	***RP1L1***	**c.5959C > T** **p.Q1987X**	het	145	150	yes, did not co-segregate	pass to whole exome sequencing

**CIC01312**, **F795**	adCD with post-receptoral defects	*RHO, PDE6B*, *GNAT1 *adRP chip	***CUBN***	**c.127C > T** **p.R43X**	het	139	102	yes, did not co-segregate	pass to whole exome sequencing
				
			***CUBN***	**c.9340G > A** **p.G3114S**	het	61	44	yes, did not co-segregate	
				
			***GUCY2D***	**c.1499C > T** **p.P500L**	het	41	34	yes, did not co-segregate	
				
			***TRPM1***	**c.3904T > C** **p.C1302R**	het	102	99	yes, did not co-segregate	

**CIC03225**, **F1362**	arRP consang.	arRP chip	***PROM1***	**c.314A > G** **p.Y105C**	het	120	115	yes, but no additional mutation	no homo, no compound hets, pass to whole exome sequencing
				
			***GUCY2D***	**c.2917G > A** **p.V973L**	het	6	2	false positive, not found by Sanger	
				
			***DSCAML1***	**c.592C > T** **p.R198C**	het	70	81	yes, but no additional mutation	
				
			***TBC1D24***	**c.641G > A** **p.R214H**	het	27	12	yes, but no additional mutation	
				
			***TMEM67***	**c.1700A > G ** **p.Y567C**	het	80	58	yes, but no additional mutation	

**CIC04757** **F2364**	Index and affected sister early onset arCD, macro-cephaly and mental retardation in affected sister consang.	-	***IMPG2***	**c.3439C > T** **p.P1147S**	homo	0	140	no	Polyphen and Sift benign, not conserved
			
			***PKD2L1***	**c.1027C > T** **p.R343C**	het	63	68		
				**c.1202T > G** **p.V401G**	het	25	19		appeared also het in 11 of our samples appeared also het in affected sister but no other mutation in less covered exons
			
			***DFNB31***	**c.1943C > A p.S648Y**	het	7	7	yes	affected sister also both variants but both come from father, no other variant in lower covered region.
				**c.2644C > A** **p.R882S**	het	27	14	yes	
			
			***EYS***	**c.7597A > G** **p.K2533E**	het	151	149	yes	Affected sister does not carry this variant
			
			***RPGRIP1***	**c.2417C > T** **p.T806I**	het	138	132	no	not conserved

**CIC04152, F1955**	male x-linked cCSNB, has affected nephew	*NYX *	***TRPM1***	**c.470C > T** **p.S157F**	het	118	130	yes, no other het mutation.	x-linked inheritance and phenotype verification

Index patients and respective gene defect are highlighted in bold. In some cases also family members were used for NGS.

## Discussion

By using NGS in 254 known and candidate genes we were able to detect known and novel mutations in 57% of families tested. In order to achieve this goal, we applied a rigorous protocol (Figure 1). To our knowledge, this is the first report using NGS to investigate all inherited retinal disorders at once. In a study restricted to adRP, Bowne and co-workers used a similar approach including 46 known and candidate genes for adRP [18]. All their cases had previously been screened and excluded for most of the known genes underlying adRP. The authors were able to identify known or novel mutations in five out of 21 cases in genes not included in a pre-screening [18]. This added five patients to their adRP cohort with known gene defects, indicating that 64% of their patients show known mutations with new genes still to be discovered in the remaining 36%. The current study provides a more exhaustive tool, since it incorporates screening of 254 genes implicated in various retinal disorders of different inheritance patterns and additional candidate genes for these phenotypes. With this approach a cohort of both pre-screened and unscreened samples, was investigated. The mutation detection rate of 57% is high and was never obtained before by high throughput screening methods. Furthermore, this approach is probably less time consuming and expensive than existing methods such as direct sequencing of all known genes or microarray analysis. Of note however is one of the variants detected with the NGS approach (i.e. p.V973L exchange in *GUCY2D*), which was not confirmed by direct Sanger sequencing, suggesting the possibility of false positive using the high throughput screening. Verification by direct Sanger sequencing of most likely pathogenic variants is therefore essential to validate NGS data, although the false positive rate is assumed to be low (in our study 1/28 verified sequence variants represented a false positive).

Overall, the study of 20 subjects from 17 families by NGS showed that most of the targeted regions are well covered (more than 98%). However, some of the regions showed a lower coverage (GC-rich regions) or were not captured (repetitive regions). This was for instance the case for two genes underlying cCSNB, (i.e. *NYX *and *GRM6*) and the repetitive region of ORF15 of *RPGR*. For GC-rich regions the capture design could be improved in the future by modifying NGS chemistry, as it was successfully achieved for Sanger sequencing using different additives, which improved the amplification and subsequent sequencing. If repetitive regions like ORF15 of *RPGR *remain problematic for sequencing by NGS, direct Sanger sequencing of these targets might be the first screening of choice; in particular for disorders caused only by a few gene defects such as CSNB, and xl-RP.

By applying NGS sequencing to our retinal panel, known and novel mutations were detected in different patients. We believe that our diagnostic tool is particularly important for heterogeneous disorders like RP, for which many gene defects with different prevalence have been associated to one phenotype. It also allows the rapid detection of novel mutations in minor genes which are often not screened as a priority by direct Sanger sequencing. This was the case in our study for three individuals from one family with adRP in which NGS detected a novel *PRPF8 *mutation in both affected and one unaffected family member (Table 4, Figure 4). In this family, the RP phenotype is mild and therefore it is possible that the unaffected member may develop symptoms later in life or alternatively it may be a case of incomplete penetrance as reported for another splicing factor gene, *PRPF31 *and recently for *PRPF8 *as well [19-22]. Interestingly, a novel *TRPM1 *mutation was identified in a patient with adCSNB, a gene previously only associated with arCSNB [23-26]. This is the first report of a *TRPM1 *mutation co-segregating with ad Schubert-Bornschein type complete CSNB. Since the location of this mutation is not different compared to other mutations leading to arCSNB, it is not quite clear how *TRPM1 *mutations might lead to either ad or arCSNB. Functional investigations are needed to validate the pathogenicity of this variant. Furthermore, this finding suggests that *TRPM1 *heterozygous mutation carriers from arCSNB families should be investigated by electroretinography to determine whether they display similar retinal dysfunction as in affected members of the presented adCSNB family. Detection of a novel *RPGR *splice site mutation in family 146 presented a challenge. The actual disease causing change was concealed under a wrongly annotated rs62638633, which had previously been clinically associated to RP by a German group http://www.ncbi.nlm.nih.gov/sites/varvu?gene=6103&rs=62638633, (personal communication, Markus Preising). These observations indicate that the stringent filtering we applied initially can mask those referenced disease causing variants. Bearing this in mind one can still first investigate unknown variants, but should then examine dbSNP for referenced variants either described to be disease causing, having a low minor allele frequency or present in interesting candidate genes. An accurate discrimination of non-pathogenic polymorphisms versus disease causing polymorphism in SNP databases is warranted to resolve this challenge.

In six families from the investigated cohort the disease causing mutations still remain to be identified. In the Stargardt patient with no pathogenic *ABCA4 *mutations two variants in *CFH *were detected, one of which (rs1061170) had previously been reported to predispose to age related macular degeneration (AMD) [27-29]. The second *CFH *change is a novel variant, affecting a highly conserved residue, not found in NGS data from the other 19 samples and never associated with a disease. The variants co-segregated in the only available family members, which were the patient's parents. Apart from the association with AMD, *CFH *mutations have been previously associated with renal diseases, the most common being membranoproliferative glomerulonephritis and hemolytic uremic syndrome, which can be also associated with an eye phenotype [30,31]. No renal dysfunction was present in our patient. To validate if the two variants identified in *CFH *are indeed disease causing, the DNA samples from other available family members for co-segregation analysis as well as characterization of functional consequences of the novel variant are needed. One patient with complete CSNB had an affected nephew and thus x-linked inheritance was assumed. However, neither Sanger nor NGS detected a mutation in the only known x-linked gene, *NYX*, causing cCSNB. To exclude recessive inheritance *TRPM1 *and *GRM6 *were investigated in detail. Indeed the patient carried a novel heterozygous *TRPM1 *variant, which affects a highly conserved amino acid and was not identified in the other 19 samples investigated here (Table 6). However, direct Sanger sequencing of lower covered regions did not identify a second mutation in this gene. Similarly no mutations in *GRM6 *were identified. These findings outline the need for additional family members to determine, through co-segregation, the pathogenicity of the numerous variants identified by NGS. This was also true for two other families with nonsense mutations in *CUBN *(Fam795) and *RP1L1 *(Fam761) (Table 6). The nonsense mutation in *CUBN*, co-segregated with the phenotype in most of the family members (Figure 5). Had we not had access to additional family members, we might have retained this gene defect as the underlying cause for adCD and considered *CUBN *as a new gene involved in adCD. None of the other putatively pathogenic mutations identified in *CUBN, TRPM1 *and *GUCY2D *co-segregated with the phenotype in this family (Table 6, Figure 5). *RP1L1 *was already a candidate for adRP [32] but was previously associated with occult macular dystrophy [33]. In our study, this variant did not co-segregate with the phenotype in other affected family members (data not shown).

This NGS study ended with six genetically unresolved families, which can be further investigated with whole exome sequencing. Although, no clear information about the actual percentage of missing gene defects underlying each group of inherited retinal disorders exists, previous studies have reported that in many cases the genetic cause still needs to be determined [18,34]. Whole exome sequencing approaches allow the detection of both, novel and known gene defects, but also generate numerous variants and therefore require the inclusion of more than one DNA sample for each family to rapidly exclude non-pathogenic variants. Due to the higher costs of exome sequencing for one sample compared to targeted sequencing, we propose to initially perform targeted sequencing in the index patient and proceed only after exclusion of a known gene defect to whole exome sequencing.

## Conclusions

In summary, our diagnostic tool is an unbiased time efficient method, which not only allows detecting known and novel mutations in known genes but also potentially associates known gene defects with novel phenotypes. This genetic testing tool can now be applied to large cohorts of inherited retinal disorders and should rapidly deliver the prevalence of known genes and the percentage of cases with missing genetic defect for underlying forms of retinal disorders.

## List of abbreviations

ad: autosomal dominant; ar: autosomal recessive; as: asymptomatic; het: heterozygous; homo: homozygous; hemi: hemizygous; - not noted; consang.: consanguinity was reported; n.a.: not applicable; CSNB: congenital stationary night blindness; RP: retinitis pigmentosa:

## Competing interests

The authors declare that they have no competing interests.

## Authors' contributions

IA was involved in the study design, participated in the choice of genes, interpreted the NGS data, clinically investigated patients, collected DNA samples, and has been involved in drafting the manuscript. KB participated in the choice of genes, interpreted the NGS data and has been involved in drafting the manuscript. TL was involved in the study design, participated in the choice of genes and has been involved in drafting the manuscript. SM-S clinically investigated patients and collected DNA samples. M-EL confirmed the NGS data by Sanger sequencing, performed control and co-segregation analysis. AG extracted DNA, confirmed the NGS data by Sanger sequencing, and performed control and co-segregation analysis. AA extracted DNA, confirmed the NGS data by Sanger sequencing, and performed control and co-segregation analysis. CM confirmed the NGS data by Sanger sequencing, and performed control and co-segregation analysis. J-PS performed NGS. ML performed the bioinformatic interpretation of NGS. J-AS clinically investigated patients and participated in the study design. SSB participated in the study design and has been involved in drafting the manuscript. CZ has made the study design, participated in the choice of genes, interpreted the NGS data and wrote the manuscript. All authors read and approved the final manuscript.
